# Abnormal resting state activity of left middle occipital gyrus and its functional connectivity in female patients with major depressive disorder

**DOI:** 10.1186/s12888-018-1955-9

**Published:** 2018-11-26

**Authors:** Changjun Teng, Jing Zhou, Hui Ma, Yarong Tan, Xin Wu, Chengbin Guan, Huifen Qiao, Jijun Li, Yuan Zhong, Chun Wang, Ning Zhang

**Affiliations:** 1 Nanjing Brain Hospital Affiliated to Nanjing Medical University, Nanjing, 210029 People’s Republic of China; 2 Nanjing PuKou Central Hospital, Nanjing, 211800 People’s Republic of China; 3 School of Psychology, Nanjing Normal University, Nanjing, 210024 People’s Republic of China

**Keywords:** Major depressive disorder, Female, Resting-state fMRI, Amplitude of low frequency fluctuations, Functional connectivity, Left middle occipital gyrus

## Abstract

**Background:**

Women are more susceptible to major depressive disorder (MDD). A possible explanation is that women have a trait tendency to engage in a ruminative response style. Depending on cognitive model of depression, attention bias, memory bias and self-referential bias were closely related among depressed patients. Previous studies have explored the neural mechanism of the cognitive biases by using amplitude of low frequency fluctuations (ALFF) or functional connectivity (FC), and few combined these two metrics, especially focusing on female patients.

**Methods:**

We assessed 25 female patients diagnosed with MDD and 13 well matched healthy controls (HCs) using Rs-fMRI. Two metrics ALFF and FC based on abnormal ALFF were explored and made comparisons.

**Results:**

Compared with HCs, female patients with MDD showed that one cluster with significantly decreased ALFF in the left middle occipital gyrus(L-MOG). Furtherly we founded depressed female subjects showed significantly lower FC between the L-MOG seed and left orbitofrontal cortex, and significantly higher FC between the L-MOG seed and left medial prefrontal gyrus and left hippocampus.

**Conclusions:**

Our results showed L-MOG may act as a connection, which involved in the processing of cognitive biases of MDD by connected with limbic-cortical regions in resting state. These findings may enhance the understanding of the neurobiological mechanism in female patients with MDD.

## Background

Major depressive disorder (MDD) is a very common heterogeneous mental disorder characterized by depressed affective and cognitive disturbances, especially for women, with an incidence and prevalence twice that of men [1]. An availably possible explanation is that women have a trait tendency to engage in a ruminative response style characterized by a repetitive pattern of self-referent cognitions [2]. Some studies furtherly posited that rumination is clearly related to attentional bias. Review found that participants who primarily respond to a negative mood by ruminating showed difficulty disengaging from negative material even after controlling for depression severity [3]. Researchers consider that female’s tendency to habitually ruminate in response to a negative mood suggests that gender will evidence greater attentional biases for negative information, because they will exhibit difficulties inhibiting negative stimuli, and that females may be more vulnerable to attentional manipulations for negative stimuli than males [4].

According to cognitive model of depression, attention, memory, thoughts and rumination biases are stable abnormalities of depressed patients. Negative bias in the processing of information plays a critical role in influencing the onset, maintenance and recurrence of depressive episodes [5, 6]. Although the causative link between attention bias and mood disorder is not completely clear, some studies have found that attentional bias modification had been used to reduce anxiety and depressive symptoms [7–9]. A meta-analysis has found reducing negative bias could decrease depressive symptoms and increasing negative bias could increase depressive symptoms [9]. These findings indicated that attentional bias may contribute directly to depressive symptoms and not a consequence of depression [10]. Some studies also suggest that attention bias arise prior to the onset of a disorder, representing a risk factor [11–13].

Attention bias have been reported to be particularly prominent of emotional face, and these findings are all from specific task state studies like identifying emotional face [14]. Task stimuli help to explore the specific stimuli process, and could not delineate a comprehensive process in common daily life. MDD is a pervasive disorder and has an impact on very realm of daily life. Biased cognitive processes play a crucial role in social function including interpersonal problems. “Resting-state hypothesis” of MDD considered neural predisposition at rest as a fundamental neural mechanism of MDD. So, study using resting state combining with functional magnetic resonance imaging (Rs-fMRI) without any task stimulus also may provide some insight for neural mechanism of pervasive abnormal cognition patterns [15].

Rs-fMRI has been widely explored in patients with the neuropsychiatric disorder, including MDD. The amplitude of low-frequency fluctuation (ALFF) is to examine low frequency fluctuation at Rs-fMRI. ALFF is confirmed to be reliable and sensitive measure in the study of both healthy and clinical populations [16–18]. Functional connectivity (FC) measures the correlation of low frequency fluctuation between spatially independent regions. MDD is considered a disease from localist models to the circuit or network models [19–21]. Numbers of studies have provided lots of findings in MDD using these two metrics. Yao and his colleagues found that male MDD patients showed higher ALFF values in the left postcentral gyrus, the left inferior parietal lobule and right precuneus and lower ALFF values in left superior temporal pole, right superior/middle frontal gyrus and bilateral crus 1 of the cerebellum for main effects of gender compared with females [22]. Buchanan investigated whole brain FC in MDD and found that depressed female patients showed significantly decreased FC in the right/left frontoparietal regions and language networks compared to healthy control subjects [23]. Another study founded that high-risk female adolescents had decrease FC between right inferior prefrontal cortex and other critical nodes of attention control network compared with low-risk ones, and they concluded that adolescent daughters might inherit depression vulnerability from their depressed patients [24]. Previous studies have also described sex difference in brain structure and function. Morphometric study in normal adults found that differences of some indices of corpus callosum and ventricles in female and male populations [25]. Amygdala volumes in adolescents showed sex differences, smaller left amygdala volumes were associated with better parental reports of emotional controls in girls and larger left amygdala volumes in boys [26]. Using fMRI, McRae explored the gender differences in cognitive reappraisal strategy of emotional regulation, and found women showed larger increases activation in prefrontal regions, smaller decreases activation in the amygdala and larger engagement of ventral striatal regions compared with men [27]. A recent multimodal meta-analysis identified abnormalities in regional cerebral blood flow and ALFF in the left insula in depressed individuals and founded that the percentage of female participants was negatively associated with the regional cerebral blood flow [28]. All of these studies showed us that gender factor should be taken into consideration in the study of fMRI. Consequently, study directly focusing on women may help to investigate potentially additional insight into the neural mechanisms in MDD.

Depending on the current most consistent theories of limbic-cortical dysfunction in MDD, MDD is accompanied by functional and structural abnormalities in many regions including the prefrontal cortex (lateral and medial) and limbic areas (such as hippocampus and amygdala) [29]. Regions in medial prefrontal cortex (mPFC) [5, 30–32] and hippocampus [5, 33–36] are believed to be the neural correlate of self-referential thoughts, rumination and biased memory, and orbitofrontal cortex (OFC) [37–39]is believed to involve in the inhibitory control and selective attention.

We are aware of few studies combining these two methods to examine resting-state differences in depressed female patients directly. Previous studies of FC usually took region of interest selection based on accumulated priori knowledge of disorder. This method has a certain subjectivity and artificial. Thus, combining ALFF and FC based on the abnormal ALFF maybe provide some insights into the neural basis of disease in term of fMRI signal of low-frequency fluctuation. We aimed to test the hypotheses that 1) female MDD would have altered ALFF activity in cortical regions related to attentional bias which acted as a connection and 2) abnormal ALFF-based FC with hippocampus and mPFC which involved in cognitive bias of depression.

## Methods

### Participants

Thirty-eight subjects participated in the present study. Twenty-five female depressed patients were recruited from the Department of Medical Psychology, Nanjing Brain Hospital Affiliated to Nanjing Medical University. Two experienced psychiatrists made a diagnosis of current depressive episode according to the structured clinical interview for DSM-IV Axis I Disorder (SCID). The patient must meet the inclusion criteria of the first episode depression, drug-naive, duration of depression less than a year, 20 < age < 50 years old, right-handed and no history of unstable cardiac or neurological disease. The exclusion criteria included schizophrenia, bipolar and any psychotic disorder, history of head injury or loss of consciousness, history of substance abuse, contraindications to MRI and 24-item Hamilton Depression Rating Scale (HAMD) score less than 18.

Thirteen right-handed aged-matched and education-matched healthy controls (HCs) with HAMD less than 8 were enrolled from the community through advertisements. The HCs group was interviewed by SCID non-patient edition to confirm without any neurological disease, mental illness, or family history of psychiatric disorders, contraindications to MRI.

### Data acquisition

All Rs-fMRI data were obtained with a 3.0 T Siemens MR scanner (Erlangen, Germany). Before scanning, a foam pad was used to minimize the head motion of all participants. Participants were instructed to keep head motionless, keep their eyes closed and not think anything till the end. Firstly, T1-weighted images were acquired to make sure that there are no brain structural changes. The parameters of three-dimensional T1-weighted sequence: repetition time 1900 ms with echo time 2.48 ms, flip angle 9°, 176 slices, 256*256 image matrix, the field of view 256*256, slice thick 1 mm with slice gap 0.5 mm. Total acquire time is 4 min 18 s. Secondly, Rs-fMRI data were acquired with single shot echo planner imaging (EPI) sequence using the parameters: repetition time 3000 ms with echo time 40 ms, flip angle 90°, 32 slices, 64*64 image matrix, the field of view 24*24 cm2, slice thick 4 mm with slice gap 4 mm. Total scan time is 5 min 06 s.

### fMRI data preprocessing

Special fMRI software Data Processing Assistant for Resting-State fMRI (DPARSF, v2.2, http://www.restfmri.net) was used to preprocess Rs-fMRI data. First six volumes were discarded to establish steady-state signal equilibrium and account for participants’ adaptation to circumstance. The remaining volumes were sliced timing and realigned head motion correction. Two HC were excluded due to excessive head motion (more than 1.5 mm or 1.5° during scanning). Furtherly, there was no significant difference between group (two sample *t* test, *t* = 0.625, *P* = 0.536 for translation, and *t* = 0.452, *P* = 0.653 for rotation) based on the formula [40]. The imaging was then spatially normalized to the standard Montreal Neurological Institute (MNI) EPI template. Spatial smoothed with 4 mm full width at half maximum Gaussian filter were done. Finally, linear trend subtraction and temporal bandpass filtering (0.01–0.08HZ) were performed to remove low-frequency drifts and physiological noise.

### ALFF calculation

ALFF was calculated and compared by the Resting State fMRI Data Analysis Toolkit (REST, V1.8, http://www.restfmri.net). Briefly, the time series was converted to the frequency domain using a fast Fourier Transformation, the square root of measures power spectrum was computed and then the mean was calculated across 0.01–0.08 Hz for each voxel. The mean square root was referred to ALFF. Each voxel was further standardized by divided by the global mean value for subsequent statistical analysis.

### FC analysis

Seed-based FC analysis was also calculated with REST. The clusters of ALFF that appeared in group differences were used as regions of interest for the FC analysis based on literature [41]. The mean time series of abnormal ALFF region was calculated as the reference time course. Then we conducted a correlation analysis between seed reference time course and the rest voxels in the whole brain. Finally, the correlation coefficients were transformed to z-value by applying Fisher’s r-to-z conversion to improve their Gaussian distribution for subsequent FC group comparisons.

### Statistical analysis

Independent sample *t*-test was used to compare demographic and HAMD scores in SPSS. Then we first do one-sample *t*-tests in MDD and HCs group using standardized ALFF that was significantly greater than 1 in RESR to make a mask for subsequent two-sample *t* test for ALFF, because of the processing of standardized by the global mean value. Differences in ALFF were assessed used two- sample *t*-tests with REST within the mask. Differences in FC were also assessed as the same with ALFF within the whole brain mask. In all the analysis, Monte Carlo simulation was applied to correct for multiple comparisons using the REST Alphasim program [42, 43], and the significance threshold of *p* < 0.05 was set by using a combination of each voxel threshold *p* < 0.05 and cluster size of 42 voxels for ALFF and 85 voxels for FC calculation.

## Results

### Demographics

Compared to HCs, there was no significant difference between the two group in terms of age or level of education. As expected, patients with MDD had significantly higher HAMD scores (Table 1).

**Table 1:** Demographic, clinical characteristics

	HCs (*n* = 13)	MDD(*n* = 25)	*t* values	*P* values
Age (years)	38.23 ± 10.12	35.80 ± 8.08	0.84	0.41^a^
Education (years)	13.92 ± 3.40	13.10 ± 2.80	0.83	0.41^a^
HAMD	1.93 ± 0.96	25.67 ± 5.31	−15.92	0.00^a^
During of disorder (months)	–	6.33 ± 3.45	–	–

*Abbreviation*: *HCs* healthy controls, *MDD* major depressive disorder, *HAMD* Hamilton Rating Scale for Depression

^a^The *P* values were obtained by two sample *t-*test

### The ALFF group results of the two group from one sample *t*-tests

**Fig. 1:**
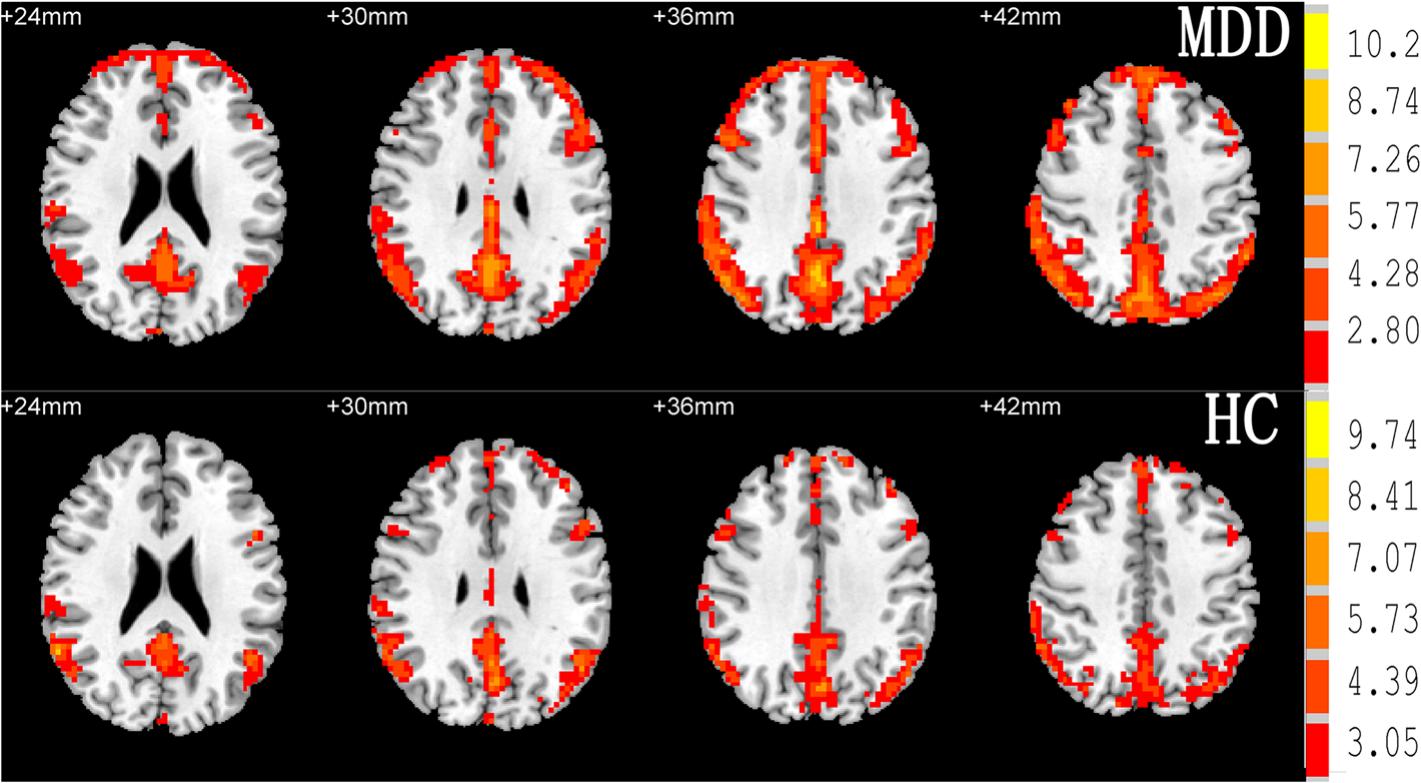
Maps of within condition patterns of resting state amplitude of low-frequency fluctuation in group MDD and HCs. The numbers above the imaging refer to the MNI z coordinate. The color bar on the right side refers to the range of *T* values

One sample t-tests reveal that bilateral frontal lobe, bilateral temporal lobe, bilateral parietal lobe, bilateral occipital lobe, anterior cingulate cortex, posterior cingulate cortex/ precuneus had a standardized ALFF value that was significantly greater than 1 in the two groups (Fig. 1).

### ALFF differences between groups from two sample *t*-tests

**Fig. 2:**
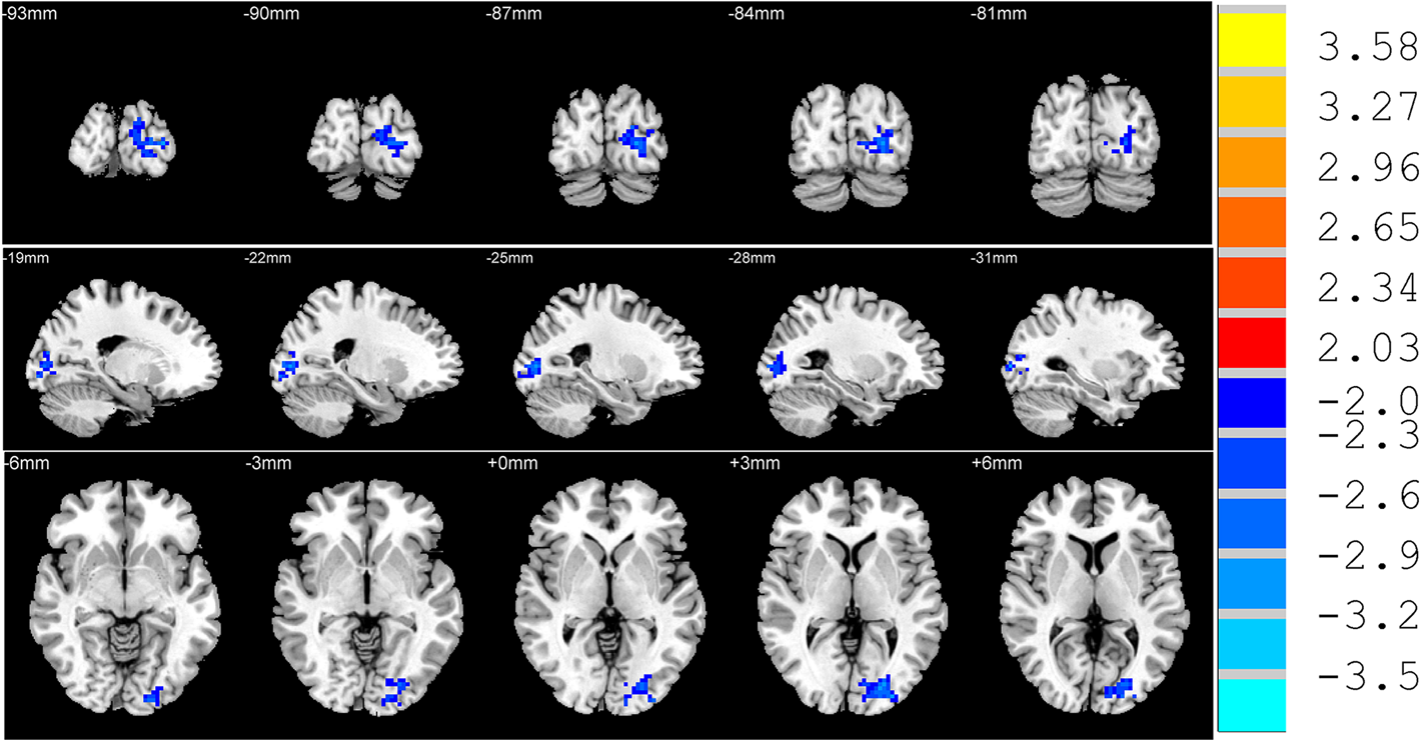
T-statistical map region showing decreased (blue) amplitude of low-frequency fluctuation(ALFF) in female patients with major depressive disorder MDD compared with healthy controls. Significant clusters of bigger than 42 voxels with correction for multiple comparison applied at *p*<0.05 (cluster-corrected with alphasim). The Numbers above the imaging refer to the MNI z coordinate. The color bar on the right side refers to the range of *T* values

Female with MDD group had only one cluster with significantly lower ALFF in left middle occipital gyrus (L-MOG) compared to HCs. There was no significantly higher ALFF cluster in MDD group (Table 2 and Fig. 2).

**Table 2:** Area of decreased amplitude of low frequency fluctuation (ALFF) and altered functional connectivity (FC) in female subjects with major depressive disorder compared to healthy controls

Metrics	Brain region (BA area)	Cluster size	MNI coordinates			*T* values
			X	Y	Z	
ALFF	Left Middle Occipital Gyrus (BA37)(L-MOG)	159	−30	−93	3	−2.90
L-MOG seed FC	Left Medial Prefrontal Gyrus(BA9/10)(L-mPFG)	115	−6	57	18	3.44
	Left Hippocampus (BA21)	112	−33	−33	−9	5.09
	Left Orbitofrontal cortex (BA11/47) (L-OFC)	85	−12	33	−27	−4.14

*Abbreviations*: *BA* Brodmann area, *MNI* Montreal Neurologic Institute

### FC differences between groups from two sample *t*-tests

**Fig. 3:**
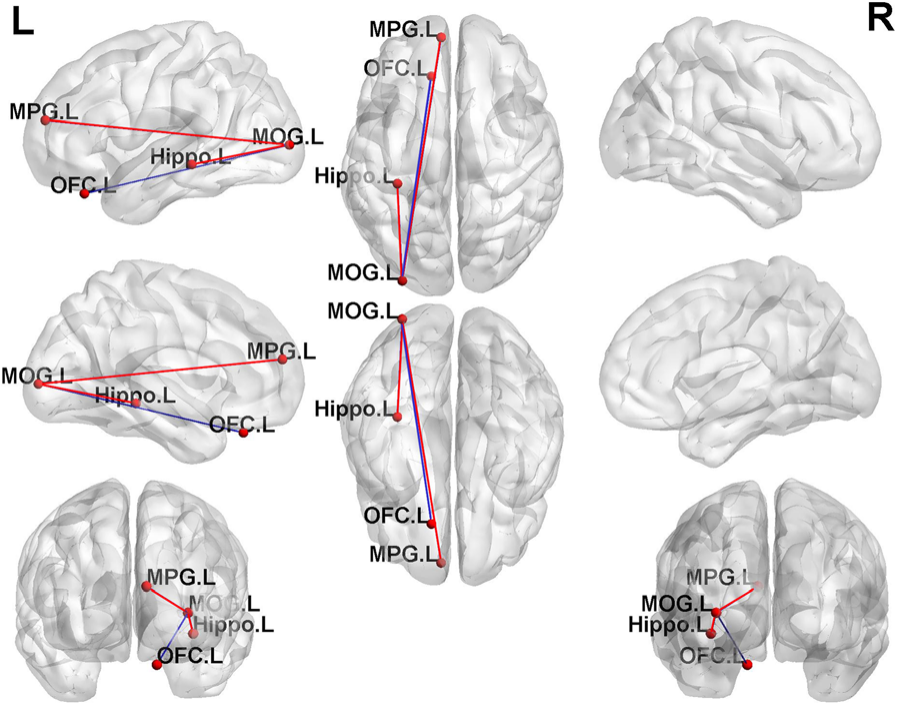
Graph visualization of functional connectivity comparison differences. Compared with healthy controls, female major depressive disorder patients show significantly increased FC of left middle occipital gyrus (MOG.L) with left medial prefrontal gyrus (MPG.L) and left hippocampus (Hippo.L) and significantly decreased FC between L-MOG and left orbitofrontal cortex (OFC.L) L, Left. The red line refers to higher FC, and the blue line refers to lower FC

The L-MOG was selected as a seed for FC analysis as it appeared between-group differences. Female MDD group showed significantly increased FC between L-MOG and left medial prefrontal gyrus (L-mPFG) and left hippocampus compared with HCs. They also showed significantly decreased FC between L-MOG and left OFC (L-OFC) compared with HCs (Table 2, Fig. 3).

## Discussion

The current study aimed to explore the neural basis of MDD in first-episode untreated female patients. We combined ALFF with FC analysis methods based on brain coactivation mechanisms among the different regions. Specifically, we sought to examine FC pattern of a region which had abnormal ALFF activation. We found that a unique decreased abnormal activation in L-MOG in depressed female patients. Moreover, significantly increased FC of L-MOG with L-mPFG and left hippocampus, and significantly decreased FC between L-MOG and L-OFC were also showed in depressed female patients.

We found significantly decrease ALFF in L-MOG which was consistent with our hypothesis. The occipital lobe contains most of the anatomical region of the visual cortex and contributes to visual information processing and communication with the cerebral cortex, and plays a role in the perception of facial emotion. Cerullo noticed that patients with bipolar-I showed decreased activation in the bilateral MOG compared with HC and MDD, while MDD only showed decreased activation in L-MOG compared to bipolar-I patients under the emotional imaging stimulus [44]. Furey found baseline activation of MOG may predict treatment response to scopolamine [45]. Many resting state studies and recent meta-analysis of Rs-fMRI also found decreased activation in L-MOG [46–49]. Structural research showed that occipital bending might be characteristic of MDD [50]. Guo and his colleagues have founded decreased ALFF in occipital cortex and considered it relevant to MDD and implicated disrupted visual processing in MDD [46]. One study demonstrated the role of MOG in category-selective attention modulating unconscious face/tool processing and found decreased activation in MOG under the face-selective attention during unconscious face processing [51]. Consistent with these studies, the decreased ALFF in L-MOG may provide a neural basis for disrupted visual processing in female MDD. A common cognitive feature of MDD is mood congruent processing bias that is a preference for negative emotional information [6, 52]. Some researches considered the model of selective bias in processing and interpretation for emotional information to be one of the risk factors of MDD for young people [13, 53] and that attention to negative information may maintain depression [5, 54]. Consequently, our finding may suggest that processing bias in MDD may be initiated as a perceptual visual bias, which may cause a series of cognitive and affective symptoms of MDD.

Furthermore, we found significantly increased FC between L-MOG and L-mPFG and hippocampus. The mPFG and its adjacent areas play a crucial role in depression symptomatology [46, 47, 55]. It has been confirmed that mPFG was involved in self-referential thought [30] and depressive rumination [30, 32]. Yoshimura showed that patients with depression displayed hyperactivity in mPFC during the self-referential processing of negative valence personality trait words [56] and negative emotional words [57]. Delaveau also found that antidepressant medicine in females depressed patients could bring the hyperactivity of mPFC during self-referential processing back to normal [58]. A visual search task study showed increased FC between mPFC and lateral occipital cortex, and observed white matter tracts between mPFC and lateral occipital cortex using imaging-guided diffusion tensor imaging [59]. In previous resting state studies, we have not found FC between mPFC and occipital cortex. The different findings may be related to differences in samples and medications status [60]. We speculate that the increase FC between L-MOG and L-mPFG may be related to exaggerated self-referential processing bias and depressive rumination in MDD.

Hippocampus is also a vital region in the pathogenesis of MDD [34, 61]. As a key structure of limbic circuit involved in memory formation, emotional learning and emotional regulation [62]. In particular, memory biases of negative information rely heavily on it [35, 36]. An associative and item recognition memory fMRI study showed that association recognition memory for object-color relationship led to bilateral hippocampus and parahippocampus activation and L-MOG, while old item recognition of objects showed activation in L-MOG and L-middle temporal gyrus. New item recognition of objects just showed activation in the bilateral hippocampus [63]. Emotional memory recollection involved in the hippocampus and mPFC [64]. Studies showed that sleeping could help to consolidate emotional memory by enhancing the FC between the hippocampus and mPFC [64] and promote the neural reorganization of remote emotional memory by enhancing the FC between mPFC and precuneus and occipital cortex [65]. Increasing FC between L-MOG and L-hippocampus in this study may be related to memory impairment and sleep disturbance in MDD.

Hippocampus and mPFG are also key nodes of default mode network (DMN) [66, 67]. Although DMN has been linked to episodic memory and memory consolidation in some studies [68], a study paid more attention to its function in self-generated thought and rumination during rest, especially in depression [66]. In this current study, we found increase FC between L-MOG and hippocampus and mPFG may be relevant to bias attention, bias memory and biased thought and rumination in MDD [5].

At last, we found decreased FC between L-MOG and L-OFC. OFC is regarded as a region in integrating sensory and emotional information through white matter connections with visual, auditory and limbic structures [39], and play a vital role in mood regulation, response inhibition, selective attention and reward processing in the pathophysiology of MDD [29, 37–39]. Previous study revealed decreased ALFF in OFC in patients with MDD and suggested hypofunction of emotion regulation [69]. Dynamic causal modeling was applied to explored neural mechanisms of a bottom-up and top-down process for emotional facial expression during memory formation. Xiu and his collogues found that emotion information could affect bottom-up connections from the occipital visual cortex to the OFC [70]. Lateral OFC act an important role in facilitating selective attention to modulate irrelevant emotional materials from the environment [37]. MDD patients showed increased OFC activity in attentional task asking to ignore sad words with attending to happy ones compared with ignoring happy with attending to sad [52]. In this sense, decrease FC between L-MOG and L-OFC may indicate impairment inhibited capability in response to visual information and related to dysfunctional mood regulation in female depression.

It is worth noting that we found left-lateralized cortical regions in female MDD. This finding was also in consistent with previous findings. Jiang has founded that differences in predominated lateralization of ALFF alteration between MDD and bipolar depression which meant left hemisphere for MDD and bilateral of bipolar depression [71]. Of course, these findings may be related to gender difference. In brain study, sex-related hemisphere lateralization was founded in healthy young Chinese adults. This study suggested that regional difference of gray matter density between men and women, and that functional regional homogeneity (ReHo) difference with higher ReHo in the right hemisphere for men and in left for women [72]. Our findings were also compatible with previous study. Recently, an event-related potential study suggested that female depression may be more vulnerable that male during emotional face processing with the unconscious negative cognitive bias, and considered that unconscious cognitive bias may be modulated by sex effects [73].

Some limitations should be addressed. First, the small number of participants and the imbalance in HCs might be insufficient to summarize the results to a larger population. Second, the data are cross-sectional; we cannot ensure the clinical diagnosis consistency of MDD patients because of the risk of switching to bipolar disorder. Third, although ALFF was used wildly, the true neurophysiological mechanism is still not clear. Future studies should include a larger number of participants with a longer observation period.

## Conclusions

In summary, the present study revealed that female subjects with MDD had decreased ALFF in L-MOG and increased FC between L-MOG with L-mPFG, and left hippocampus and decreased FC between L-MOG and L-OFC. Importantly, these findings indicated L-MOG act as a connection, and abnormal FC may be potentially related to cognition biases of MDD and may help to advance our understanding of the neurobiological mechanism underlying female depressed patients.

## Abbreviations

ALFF: Amplitude of low-frequency fluctuation
FC: Functional connectivity
HCs: Healthy controls
L-MOG: Left middle occipital gyrus
L-mPFG: Left medial prefrontal gyrus
L-OFC: Left orbitofrontal cortex
MDD: Major depressive disorder
mPFC: Medial prefrontal cortex
OFC: Orbitofrontal cortex
Rs-fMRI: Resting state functional magnetic resonance imaging
